# Modulating effects of plasma containing anti-malarial antibodies on *in vitro *anti-malarial drug susceptibility in *Plasmodium falciparum*

**DOI:** 10.1186/1475-2875-9-326

**Published:** 2010-11-16

**Authors:** Preeyaporn Monatrakul, Mathirut Mungthin, Arjen M Dondorp, Srivicha Krudsood, Rachanee Udomsangpetch, Polrat Wilairatana, Nicholas J White, Kesinee Chotivanich

**Affiliations:** 1 Department of Clinical Tropical Medicine, Faculty of Tropical Medicine, Mahidol University, 420/6 Rajvithi, Bangkok, Thailand 10400; 2 Department of Parasitology, Phramongkutklao College of Medicine, Bangkok, Thailand; 3 Mahidol-Oxford Tropical Medicine Research Unit, Faculty of Tropical Medicine, Mahidol University, Bangkok, Thailand; 4 Critical Care Research Unit, Department of Clinical Tropical Medicine, Mahidol University, Bangkok, Thailand; 5 Department of Pathobiology, Faculty of Science, Mahidol University, Bangkok, Thailand

## Abstract

**Background:**

The efficacy of anti-malarial drugs is determined by the level of parasite susceptibility, anti-malarial drug bioavailability and pharmacokinetics, and host factors including immunity. Host immunity improves the *in vivo *therapeutic efficacy of anti-malarial drugs, but the mechanism and magnitude of this effect has not been characterized. This study characterized the effects of 'immune' plasma to *Plasmodium falciparum*on the *in vitro *susceptibility of *P. falciparum *to anti-malarial drugs.

**Methods:**

Titres of antibodies against blood stage antigens (mainly the ring-infected erythrocyte surface antigen [RESA]) were measured in plasma samples obtained from Thai patients with acute falciparum malaria. 'Immune' plasma was selected and its effects on *in vitro *parasite growth and multiplication of the Thai *P. falciparum *laboratory strain TM267 were assessed by light microscopy. The *in vitro *susceptibility to quinine and artesunate was then determined in the presence and absence of 'immune' plasma using the ^3^H-hypoxanthine uptake inhibition method. Drug susceptibility was expressed as the concentrations causing 50% and 90% inhibition (IC_50 _and IC_90_), of ^3^H-hypoxanthine uptake.

**Results:**

Incubation with 'immune' plasma reduced parasite maturation and decreased parasite multiplication in a dose dependent manner. ^3^H-hypoxanthine incorporation after incubation with 'immune' plasma was decreased significantly compared to controls (median [range]; 181.5 [0 to 3,269] cpm versus 1,222.5 [388 to 5,932] cpm) (*p*= 0.001). As a result 'immune' plasma reduced apparent susceptibility to quinine substantially; median (range) IC_50 _6.4 (0.5 to 23.8) ng/ml versus 221.5 (174.4 to 250.4) ng/ml (*p *= 0.02), and also had a borderline effect on artesunate susceptibility; IC_50 _0.2 (0.02 to 0.3) ng/ml versus 0.8 (0.2 to 2.3) ng/ml (*p *= 0.08). Effects were greatest at low concentrations, changing the shape of the concentration-effect relationship. IC_90 _values were not significantly affected; median (range) IC_90 _448.0 (65 to > 500) ng/ml versus 368.8 (261 to 501) ng/ml for quinine (*p *> 0.05) and 17.0 (0.1 to 29.5) ng/ml versus 7.6 (2.3 to 19.5) ng/ml for artesunate (*p *= 0.4).

**Conclusions:**

'Immune' plasma containing anti-malarial antibodies inhibits parasite development and multiplication and increases apparent *in vitro *anti-malarial drug susceptibility of *P. falciparum*. The IC_90 _was much less affected than the IC_50 _measurement.

## Background

Falciparum malaria remains the most important parasite infection in the tropical world. Development of anti-malarial drug resistance is a major threat for malaria control. Early signs of low-grade resistance can be obscured by anti-malarial immunity. The positive contribution of host immunity to the therapeutic response to anti-malarial drugs has been recognized for nearly a century [1]. In endemic areas, protective immunity is acquired and maintained with repeated exposure to *Plasmodium falciparum *and is an important factor determining therapeutic outcome following anti-malarial drug treatment [2,2-5]. Failing drugs can appear effective if tested in semi-immune adults, whilst cure rates in children from the same area are unacceptable [4]. The host defence against malaria, which includes pre-erythrocytic immunity, blood stage immune responses and augmented splenic clearance function, is still poorly characterized. Antibody clearly plays a role; for example increased levels of anti-MSP1_19 _IgG1 were associated with improved efficacy of sulphadoxine-pyrimethamine in Gabonese children [5] but the precise contributions of this and other antibodies to drug efficacy were not quantitated. Immunity is associated with both augmentation of parasite clearance responses and increased cure rates. An increase in parasite clearance times after treatment with artesunate-mefloquine combination therapy on the Thai-Myanmar border after 2002 coincided with a sharp reduction in malaria transmission in the area could be explained by lower immunity in this population [6]. The quantitative contribution of host immunity to drug efficacy is difficult to assess, and there are few studies addressing this *ex vivo*. The current study quantitated the effects of 'immune' plasma containing anti-malarial antibodies on parasite growth and susceptibility of *P. falciparum *to quinine and artesunate.

## Methods

### Malaria parasites

*Plasmodium falciparum *Thai laboratory strain TM267, was cultured in malaria culture medium (MCM) supplemented with 0.5% Albumax II (Gibco, New Zealand) as previously described [7]. Parasites were synchronized to ring stage through treatment with 5% D-sorbitol [8] just prior to the experiments.

### Selection of 'immune' plasma

Plasma was obtained from patients with acute *P. falciparum *malaria taking part in clinical studies performed at the Hospital for Tropical Diseases in Bangkok, Thailand approved by the Ethics committee of the Faculty of Tropical Medicine, Mahidol University. Patients who had no history of anti-malarial drug treatment before admission were screened for previous treatment with quinine and mefloquine using dipstick. Plasma was assessed for the presence of antibodies against blood stage antigens (mainly ring-infected erythrocyte surface antigen [RESA]) by an immunofluorescence assay (IFA), as described previously [9]. In brief, 10 μl of plasma (1:50 v/v in phosphate buffered saline [PBS]) was applied on an antigen-coated slide, incubated at room temperature for 30 minutes, and then washed twice with PBS. Five microlitres of rabbit anti-human IgG conjugated to fluorescein isothiocyanate (FITC; DAKO, Denmark) (1:50 v/v in PBS) was then added to each spot on the antigen-coated slide, incubated at room temperature for 30 minutes (protected from light), and then washed twice with PBS. The slides were counterstained and mounted with 10 μg/ml ethidium bromide (Sigma, USA) in 50% glycerol (Sigma, USA), and then examined under a fluorescence microscope (Model BX60; Olympus, Japan). Plasma reacting positively showed as a coated green fluorescence on the surface of infected red blood cells. Plasma positive for blood stage antibodies at a titre 1:50 (v/v) was then further assessed at titres of 1:250 and 1:1,250 (v/v). 'Immune' plasma was defined as plasma containing blood stage antibodies at titre greater than 1:50. Individual plasma (N = 1) at a titre of 1:50 was used for the assessment of the effect on the parasite growth. Plasma at tires of 1:250 (N = 2) and 1:1,250 (N = 1) were used for the assessment of the effects on the anti-malarial drug susceptibility. The plasma samples were then heated at 56°C for 30 minutes and then kept at -20°C until used.

### Effects of 'immune' plasma containing anti-malarial antibodies on growth of *Plasmodium falciparum*

Twenty-five microlitres of synchronized ring stage *P. falciparum*(TM267), at 1% *P. falciparum *infected erythrocytes with >80% ring stages were incubated with either 25 μl 'immune' plasma containing anti-malarial antibodies or plasma from healthy subjects at concentrations of 2.5%, 5%, 10%, 20%, and 40% (v/v in MCM) at 37°C for 48 hours. Parasites incubated with MCM supplemented with 0.5% Albumax II (without 'immune' plasma) were included as controls. Parasite growth and stage of development were assessed by microscopy [10]. Parasite multiplication was then assessed as the percentage of ring-infected red cells in all infected red blood cells after 48 hours incubation in the presence of 'immune' plasma containing anti-malarial antibodies relative to control (without 'immune' plasma). All experiments were repeated four times.

### Drug susceptibility assay

Assessments of parasite growth and anti-malarial drug susceptibility of *P. falciparum *TM267 were assessed by an isotopic assay using tritium [^3^H]-hypoxanthine as described previously [11]. In brief, 50 μl of a 3% haematocrit red cell suspension containing 1% *P. falciparum *infected erythrocytes with >80% in the ring stage of development, was incubated with quinine dihydrochloride (A.N.B. Laboratories Co., Ltd., Thailand) (range of concentrations: 7.80-500 ng/ml) and artesunate (Guilin Pharmaceutical Co., Ltd., China) (range of concentrations: 0.16-10 ng/ml), either in the absence or presence of 10% (v/v) 'immune' plasma containing anti-malarial antibodies. Parasites were incubated at 37°C until development to the trophozoite stage. ^3^H-hypoxanthine (25 μl: 0.025 uCi/μl) was then added to each well after which the plates were incubated at 37°C and 5% CO_2 _for 24 hours until parasites reached the mature schizont stage, and then frozen at -80°C. After thawing of the sample, the lysed cells were transferred to a glass fibre filter (Wallac, Turku, Finland) using a cell harvester (Packard Instruments, Meriden, Conn.) and ^3^H-hypoxanthine uptake was measured by a scintillation counter (MicroBeta; Wallac Trilux). Four experiments were done in duplicate. The measure of drug susceptibility was the concentration of drug inhibiting ^3^H-hypoxanthine uptake by 50% and 90% relative to control (50% and 90% inhibitory concentrations; IC_50 _and IC_90_). The IC_50 _and the IC_90 _values were assessed by fitting a sigmoid dose response curve using WinNonlin software version 4.1 (Pharsight Corporation, CA).

### Statistical analyses

Statistical analyses was performed using SPSS version 11.5 (SPSS Inc., Illinois, USA). The Mann-Whitney U test was used for comparing groups with non-normally distributed data. A *p*-value ≤ 0.05 was considered statistically significant.

## Results

### The effects of 'immune' plasma on the growth of *Plasmodium falciparum*

Seventy-eight plasma samples from uncomplicated malaria patients were screened for antibodies against *P. falciparum *ring stages antigens using the immunofluorescence assay (IFA). A total of 51 patients (65%) had plasma containing blood stage antibodies assessed by IFA, of whom 31 (61%) had a titre of 1:50, 17 (33%) a titre of 1:250, and 3 (6%) a titre of 1:1,1250. In the *in vitro *culture, control plasma from healthy subjects did not react with infected red blood cells. Parasites developed completely from ring to schizont stage over a 48 hour time period. The median (range) parasitaemia after schizogony was 1.6% (1.5-1.7), 1.5% (1.4-1.6), 1.5% (1.4-1.7), 1.4% (1.3-1.4), and 1.2% (1.0-1.4) in the presence of 2.5%, 5%, 10%, 20%, and 40% (v/v) normal plasma, respectively (Table 1). Growth was significantly inhibited (*p *≤ 0.05) by concentrations of normal plasma ≥20% but not by lower concentrations. Parasite growth was inhibited more after incubation with 'immune' plasma. In the presence of 'immune' plasma (titre of 1:50) at 2.5%, 5%, 10%, 20%, and 40% (v/v), the corresponding parasitaemia was 1.7% (1.2-2.1), 1.4% (0.7-1.4), 1.1% (0.9-1.2), 1.1% (0.5-1.2), and 0.9% (0.7-1.2), respectively. The parasitaemia after incubation with 10%, 20%, and 40% 'immune' plasma were all significantly reduced compared to control (*p *< 0.05) (Table 1). Thus, the median (range) number of ring infected red cells after schizogony was reduced by 8.2% (0-26.3), 40.3% (0-70.3), 74.5% (33.3-83.1), 80% (57.1-100), and 93.9% (75-100) in the presence of 2.5%, 5%, 10%, 20%, and 40% 'immune' plasma, respectively (*p *for trend = 0.001).

**Table 1:** *Plasmodium falciparum *parasitaemia in culture in the presence of plasma from healthy donors and 'immune' plasma

Plasma concentration	Median (range) % parasitaemia after 48 hours incubation *	
	
	Normal plasma	'Immune' plasma **
Control (MCM)	1.7 (1.5-2.6)	1.7 (1.4-2.3)
2.5%	1.6 (1.5-1.7)	1.7 (1.2-2.1)
5%	1.5 (1.4-1.6)	1.4 (0.7-1.4)
10%	1.5 (1.4-1.7)	1.1 (0.9-1.2) ***
20%	1.4 (1.3-1.4) ***	1.1 (0.5-1.2) ***
40%	1.2 (1-1.4) ***	0.9 (0.7-1.2) ***

*- The percentage of infected red blood cells counted per 1,000 red blood cells.

**- 'Immune' plasma (titre of 1:50)

***- Significant difference compared to control; *p *≤ 0.05 (N = 4). MCM is malaria culture medium.

**Figure 1:**
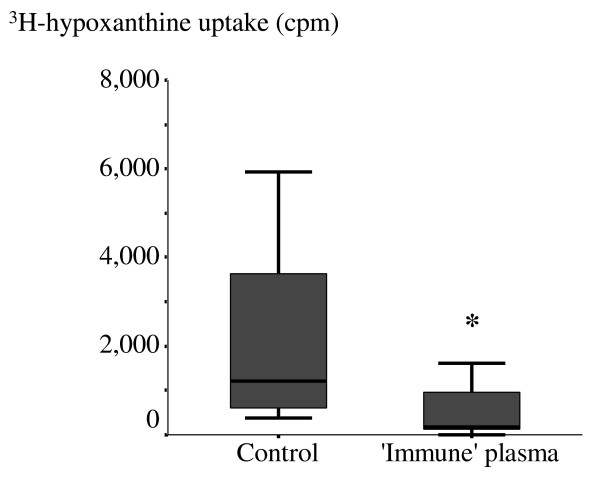
**Plasma from acute malaria inhibits ^3^H-hypoxanthine uptake by *Plasmodium falciparum***. Parasites were incubated in malaria culture medium with 10% (v/v) 'immune' plasma containing anti-malarial antibodies for 48 hours. Parasite uptake of hypoxanthine (in counts per minute; cpm) is shown as the median values with 25^th ^and 75^th ^percentiles and ranges from 4 experiments in duplicate. Control parasites were incubated in culture medium with 10% plasma from healthy donors. ^* ^*p *≤ 0.05 (Mann-Whitney U test).

The absolute ^3^H-hypoxanthine uptake after incubation with 10% 'immune' plasma was markedly lower compared to control (median [range]; 181.5 [0-3,269] cpm versus 1,222.5 [388-5,932] cpm; *p*= 0.001) (Figure 1). Thus, 'immune' plasma inhibited parasite growth and the development from ring to schizont stages and also inhibited the multiplication of *P. falciparum*.

### Effects of 'immune' plasma on the *in vitro *measurement of anti-malarial drug susceptibility

**Figure 2:**
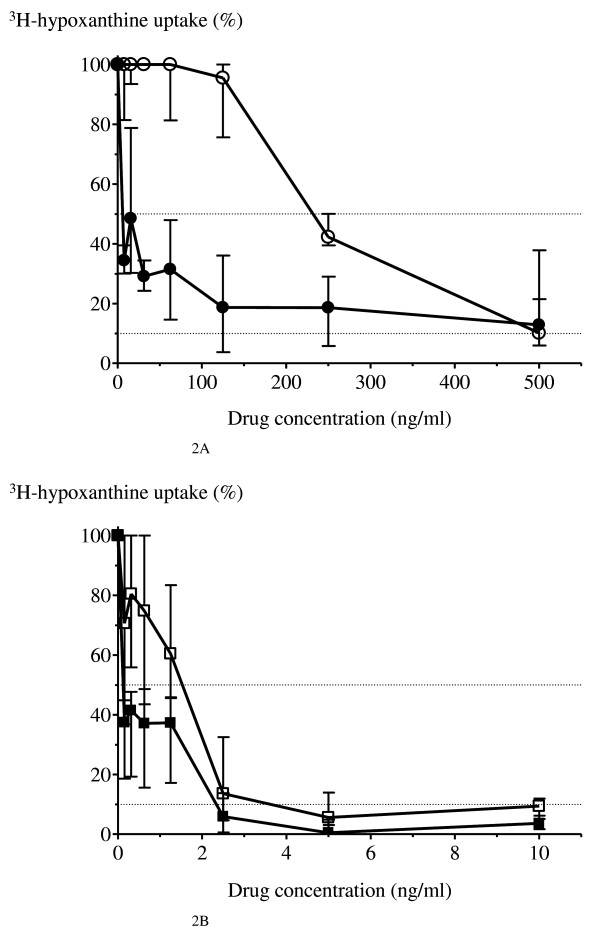
**The dose-response curve of *P. falciparum *anti-malarial drug susceptibility**. Parasites were incubated with **A) **quinine without 'immune' plasma (white circle) and quinine with 10% 'immune' plasma (black circle) (range: 7.80-500 ng/ml); and **B) **artesunate without 'immune' plasma (white square) and artesunate with 10% 'immune' plasma (black square) (range: 0.16-10 ng/ml). The percentage of ^3^H-hypoxanthine uptake was plotted against drug concentrations. The dotted lines represent 50% and 90% inhibition. Data are shown as the median values and interquartile ranges from 4 experiments.

**Figure 3:**
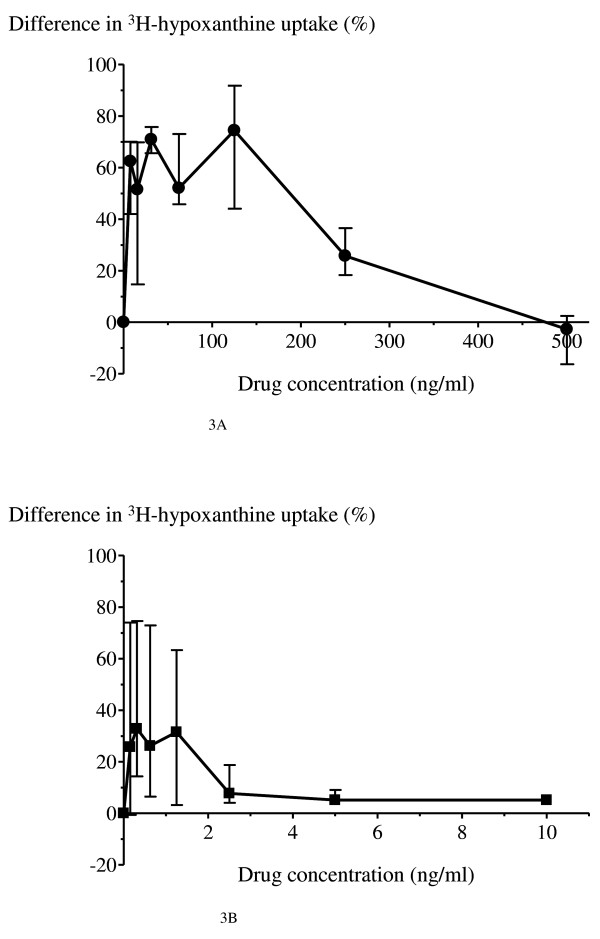
**Modulating effects of 'immune' plasma containing anti-malarial antibodies on *in vitro P. falciparum *anti-malarial drug susceptibility**. The magnitude of the effect of 'immune' plasma on the susceptibility to **A) **quinine (black circle) and **B) **artesunate (black square) expressed as the percentage of ^3^H-hypoxanthine uptake without 'immune' plasma subtracted to that of drug combined with 10% 'immune' plasma containing anti-malarial antibodies. Data are shown as the median values and interquartile ranges from 4 experiments.

The susceptibility of *P. falciparum *to the anti-malarial drugs quinine and artesunate, either in the presence or absence of 10% 'immune' plasma is shown in Figure 2. In the presence of 'immune' plasma (titres of 1:250 and 1:1,250), hypoxanthine uptake was markedly reduced at lower drug concentrations (Figure 3). The median (range) derived IC_50 _values for quinine in the presence and absence of 'immune' plasma were 6.4 (0.5-23.8) ng/ml and 221.5 (174.4-250.4) ng/ml, respectively (*p *= 0.02). The corresponding median (range) derived IC_90 _values for quinine were not significantly different; 448.0 (65->500) ng/ml and 368.8 (261-501) ng/ml, respectively (*p *> 0.05). The median (range) derived IC_50 _values for artesunate in the presence and absence of 'immune' plasma were 0.2 (0.02-0.3) ng/ml and 0.8 (0.22-2.29) ng/ml, respectively (*p *= 0.08). The corresponding median (range) derived IC_90 _values for artesunate were not significantly different: 17.0 (0.1-29.5) ng/ml and 7.6 (2.3-19.5) ng/ml, respectively (*p *= 0.4).

## Discussion

Assessing anti-malarial resistance in patients living in malaria endemic regions of the world is hampered by the presence of host immunity, which can act as an important confounder [1]. Early resistance can thus go unnoticed, because host immunity assists the drug in parasite clearance. Conversely, waning immunity in a population with a reduction in malaria transmission can cause a false impression of reduced drug efficacy. Exactly which immunological factors are important in enhancing anti-malarial drug efficacy remains unclear. The current study investigated the role of 'immune' plasma containing anti-malarial antibodies in an *in vitro *growth system and standard sensitivity drug assay. The study confirms earlier studies and the experience of malariologists conducting *in vitro *susceptibility testing that 'immune' plasma significantly inhibits the development of *P. falciparum *from the ring to schizont stage of development and also inhibits parasite multiplication. The mechanism of action of antibodies is not well understood. Antibodies are thought to reduce parasite multiplication through interference with merozoite invasion (e.g. through anti-MSP-1 [12] and anti-EBA-175 antibodies [13]) and with intraerythrocytic development [14] (e.g. through anti-RESA [15-18] and anti-PfEMP-1 antibodies [19]). How they impair growth is poorly understood. Anti-RESA antibodies have been shown previously to inhibit parasite growth [15-17] and multiplication [18] and they can be used as markers of the broader humoral immune response [20]. Anti-MSP-1 antibodies have been shown to prevent the processing of merozoite surface proteins [12] and anti-EBA-175 antibodies have been proposed to interfere with the recognition of erythrocyte ligands, both involved in the invasion process [13]. Antibodies to PfEMP-1 agglutinate infected RBCs, and through this mechanism are thought to inhibit merozoite invasion and parasite multiplication [19].

In addition to anti-malarial antibodies, plasma obtained from patients with an acute falciparum malaria infection also contains other factors which inhibit parasite growth. Acute phase proteins such as C-reactive protein, α1-acid glycoprotein, mannose-binding protein, and complement may affect the multiplication of *P. falciparum*. It has been proposed that α1-acid glycoprotein inhibits parasite multiplication by interfering with parasite-erythrocyte interactions during the invasion process [21]. C-reactive protein has been proposed to prevent the penetration of sporozoites into hepatocytes and to inhibit parasite replication during pre-erythrocytic development [22]. Mannose-binding protein (MBP) can initiate the lectin complement pathway eventually leading to cell lysis [23]. In the present study, inactivated plasma by heating at 56°C for 30 minutes was used in the experiments. Through this procedure proteins of the complement system lost their enzymatic activity [24] and therefore, complement factors and MBP were unlikely to have interfered with parasite development in the current study. The study also addressed the effects of non-specific proteins in plasma from normal donors on parasite development. Normal plasma showed no effect in concentrations up to 10% (v/v), but did inhibit parasite growth in higher concentrations of 20% and 40% (v/v). For this reason, a 10% plasma concentration (v/v) was used for the assessment of the effect of 'immune' plasma in the anti-malarial drug sensitivity assays. We found that plasma containing anti-malarial antibodies significantly increased the apparent susceptibility of *P. falciparum *to quinine and also had a borderline effect on artesunate. These effects were greater at low drug concentrations distorting the normal sigmoid shape of the concentration-effect relationship. IC_50 _values for quinine were markedly altered whereas IC_90 _values were not affected significantly. As IC_50 _values are usually reported, these findings suggest that improved malaria control with consequent population reduction in malaria antibody titers might lead to a rise in IC_50 _values of fresh isolates. This could be mistaken as indicating increasing resistance. The effects of 'immune' plasma on quinine IC_50 _values were much greater than on artesunate. The stage-specificity of the effect of anti-malarial antibodies might explain this difference. If antibodies recognize antigenic determinants on more mature trophozoites and therefore act predominantly at that stage, then they may have a greater effect in synergistically with drugs, which act only at this stage. Quinine acts predominantly on mature parasites whereas artesunate has a broad stage-specificity and acts on both young and mature stages. Future studies to characterize and assess the factors in 'immune' plasma which interfere specifically with parasite growth are required.

## Conclusions

'Immune' plasma containing anti-malarial antibodies inhibits intraerythrocytic development of *P. falciparum *as well as parasite multiplication in an *in vitro *growth system. 'Immune' plasma increased *in vitro*susceptibility to quinine and also had a borderline effect on artesunate susceptibility. Effects were greater at low drug concentrations, so the IC_50_was affected much more than the IC_90 _estimates.

## Competing interests

The authors declare that they have no competing interests.

## Authors' contributions

PM: carried out the experimental work, data analysis, and wrote the first draft of the manuscript. MM: gave technical support and helped preparing the manuscript. RU: provided laboratory support and helped in the revision of the manuscript. SK and PW: contributed to patient recruitment and patient care as well as collection of blood samples. AMD: helped in data analysis and writing of the manuscript. NJW: helped design the experiments, revise the manuscript, and analyse the data. KC: helped design the experiments, gave technical support, supervision, and helped in data analysis and writing of the manuscript. All authors have been seen and approved this manuscript.
